# Radio-detoxified LPS alters bone marrow-derived extracellular vesicles and endothelial progenitor cells

**DOI:** 10.1186/s13287-019-1417-4

**Published:** 2019-10-29

**Authors:** Hargita Hegyesi, Nikolett Sándor, Géza Sáfrány, Virág Lovas, Árpád Kovács, Angéla Takács, László Kőhidai, Lilla Turiák, Ágnes Kittel, Krisztina Pálóczi, Lóránd Bertók, Edit Irén Buzás

**Affiliations:** 1 Department of Genetics, Cell- and Immunobiology, Semmelweis University, Budapest, Hungary; 2 National Research Directorate for Radiobiology and Radiohygiene, National Public Health Center, Budapest, Hungary; 3 MS Proteomics Research Group, Research Centre for Natural Sciences, Hungarian Academy of Sciences, Budapest, Hungary; 4 Institute of Experimental Medicine, Hungarian Academy of Sciences, Budapest, Hungary; 5 MTA-SE Immune-Proteogenomics Extracellular Vesicles Research Group, Semmelweis University, Budapest, Hungary

**Keywords:** Endothelial progenitor cell, Radio-detoxified endotoxin, Extracellular vesicles, Exosomes, Bone marrow, IFITM3

## Abstract

Stem cell-based therapies raise hope for cell replacement and provide opportunity for cardiac regenerative medicine and tumor therapy. Extracellular vesicles are a membrane-enclosed intercellular delivery system with the potential to improve the therapeutic efficacy of the treatment of a variety of disorders. As the incidence of breast cancer continues to rise, radiotherapy has emerged as a leading treatment modality. Radiotherapy also increases the risk of coronary heart disease and cardiac mortality. In a chest-irradiated mouse model of cardiac injury, we investigated the effects of local irradiation. We found an increased lethality after 16 Gy irradiation. Importantly, radio-detoxified LPS (RD-LPS) treatment prolonged the survival significantly. By flow cytometry, we demonstrated that upon administration of RD-LPS, the number of bone marrow-derived endothelial progenitor cells increased in the bone marrow and, in particular, in the circulation. Furthermore, mass spectrometry analysis showed that RD-LPS altered the proteomic composition of bone marrow cell-derived small extracellular vesicles (sEVs). RD-LPS treatment increased interferon-induced transmembrane protein-3 (IFITM3) expression markedly both in bone marrow cells and in bone marrow cell-derived small extracellular vesicles. This is the first study to demonstrate that radio-detoxified LPS treatment induces an increase of circulating endothelial progenitor cells (EPCs) in parallel with a reduced radiotherapy-related mortality. While the total number of bone marrow-derived extracellular vesicles was significantly increased 24 h after treatment in the RD-LPS groups, the number of endothelial progenitor cells was reduced in animals injected with GW4896 (a chemical inhibitor of exosome biogenesis) as compared with controls. In contrast to these in vivo results, in vitro experiments did not support the effect of sEVs on EPCs. Our data raise the intriguing possibility that IFITM3 may serve as a marker of the radio-detoxified LPS treatment.

## Introduction

Stem cell therapy holds promise for tissue regeneration, but the direct replacement of cells in the damaged part of the tissue is limited and the survival of cells still remain questionable [1]. Tissue injury in the heart, lung, and bone marrow (BM) are among the most important side effects of breast cancer radiotherapy, which limits the success of tumor treatment [2–4]. However, the mechanisms through which ionizing radiation induces BM injury remains poorly understood [5]. Experimental models such as the murine radiation-induced cardiomyopathy model have proven useful to study the therapeutic approaches that mitigate radiation injury [6–8].

Several studies have demonstrated the protective effects of radio-detoxified (gamma irradiation-fragmented) lipopolysaccharide (RD-LPS), also referred to as Tolerin [9], in reducing radiation-induced tissue damage [10]. In the present study, we investigated for the first time the effects of RD-LPS in a cardiotoxicity model. We focused on endothelial progenitor cells (EPCs) and BM cell-derived small extracellular vesicles (sEVs) as potential biomarkers of the RD-LPS effect. BM-derived EPCs have been shown to have regenerative potential in cardiac diseases both in preclinical and clinical settings [11]. Recently, circulating EPCs have been suggested as sensitive and reproducible markers of endothelial dysfunction caused by ionizing radiation [12]. In addition, EPCs carry both endothelial cell markers (such as VEGFR-2 and CD31) [13, 14] and one or more stem cell antigens such as CD34 [15].

Rapidly accumulating data indicate that cells release extracellular vesicles (EVs) that mediate cell-cell communication by shuttling lipids, nucleic acids, metabolites, and proteins to recipient cells [16]. The cargo of EVs is cell- and disease-type related and confers specific features to EVs, mediating their biological functions [17–19]. In our chest-irradiated mouse model, here we investigated not only EPCs but also EVs as potential novel mediators of the effect induced by RD-LPS. In this study, the biogenesis of EVs was not addressed, and therefore, we referred to EVs based on their size only [18].

Interferon-induced transmembrane protein 3 (IFITM3) was initially described in development, apoptosis, cell proliferation, and cell signaling [20, 21]. Also, recently, it was suggested that ectopic expression of IFITM3 could restrict infection by viruses [22], by potently inhibiting EV-cell fusions. Here we addressed the question concerning the role of IFITM3 in the RD-LPS-induced EPC activation.

## Materials and methods

### Animals and irradiation

C57BL/6 mice (Charles River Laboratories) were exposed to local 16 Gy chest irradiation as described previously [23]. Mice were irradiated by a 250-kV X-ray irradiator (THX-250) using a dose rate of 1.23 Gy/min. A special lead shield was used to deliver a localized dose of radiation to the heart region only. Survival was monitored daily, and the experiment was terminated 250 days post-irradiation.

### Histology and morphometric analysis

After 250 days following irradiation, the animals were sacrificed by cervical dislocation, and the total body weight and the heart weight were measured. The hearts were excised and divided into two pieces on the vertical axis (for DNA isolation and for morphometric analysis). The tissues were fixed in formalin overnight and embedded in paraffin. Blocks were sectioned in 4-μm cross-sectioned slices and stained with Van Gieson staining to determine the extent of collagen, indicative for fibrosis [24]. The slides were photographed with Nikon Eclipse 80i, × 20 objective, 10 random fields per slide were taken into consideration. Of these sections, 4 were analyzed per heart. In total, we analyzed 160 images to evaluate a cross-sectional area of myocytes by ImageJ program [25, 26].

### Detection of the common deletion (3867 bp) in the mitochondrial genome by qPCR

Total DNA (genomic and mitochondrial together) was isolated from the heart tissues with the MasterPure DNA Purification kit (Epicenter) following the manufacturer’s instructions. DNA concentration was measured by spectrophotometry. The amount of deleted mtDNA copies was estimated by real-time PCR, relative to the nuclear GAPDH gene. The primers were the GAPDH-F: TCACCACCATGGAGAAGGC, R: GCTAAGCAGTTGGTGGTGCA and the delMT-F: TCATTCTAGCCTCGTACC AACA, R: GAGGTCTGGGTCATTTTCGTTA, respectively. QPCR reactions are carried out using the SYBR Green PCR kit (Bioline), in a Corbett RG 6000 instrument.

### Bone marrow cell apoptosis measurement

BM was isolated from the femora of mice by flushing the shaft with PBS. For apoptosis measurements, 1 × 10^6^ BMCs per animal were incubated with 3% BSA for 20 min. After washing cells once with cell culture media (RPMI-1640; Gibco), samples were resuspended in 100 μl of annexin V binding buffer (BD Pharmingen) containing Lin cocktail-APC (Miltenyi), and annexin V-FITC (Themo Fisher Scientific). Before analysis, 1 μl of Topro-3 (1 μg/ml; Invitrogen) was added to the samples. After washing them once with PBS, we measured samples by using a FACSCalibur (BD Bioscience).

### Measurement of EPC differentiation, and DiI-ac-LDL uptake

BM isolated from femurs were collected in endothelial growing media (EGM)-2 complete medium (Lonza). BMCs were seeded onto six-well plates at a final density of 1.5 × 106 cells. Twenty-four hours after seeding, non-adherent cells were removed and attached cells were further cultured at 37 °C and 5% of CO_2_. Culture media was changed every 2 days until the first EPC colonies appeared or up to 10 days. EPCs were incubated with 1,1′-dioctadecyl-3,3,3′,3′- tetramethylindo-carbocyanine-labeled Ac-LDL (Dil-ac-LDL, Life Technologies), fixed with 4% paraformaldehyde, and measured by flow cytometry (FACSCalibur, BD Bioscience), and for morphological studies, the stained samples were observed on a fluorescent microscope (Zeiss Celldiscoverer). Pictures were taken with a × 20 objective magnification.

### Isolation of small extracellular vesicles

BM cells (BMCs) were cultured in RPMI-1640 (Thermo Fisher Scientific) supplemented with penicillin (100 U/ml), streptomycin (100 μg/ml), and l-glutamine (2 mM) (all from Sigma) and 2.5% EV-free fetal calf serum (Gibco). After 24 h, the conditioned medium was centrifuged at 600*g* for 10 min to remove cells. The supernatant was filtered through a 5-μm filter then centrifuged at 2000*g* for 30 min at 4 °C. Subsequently, supernatants were filtered again through a 0.8-μm membrane and centrifuged at 12,500*g* for 30 min at 4 °C. The supernatant was next filtered through a 0.2-μm filter and pelleted at 100,000*g* for 70 min at 4 °C using an MLA-55 rotor in an Optima Max XP ultracentrifuge (Beckman-Coulter). The small EV (sEV)-enriched pellet was washed once with PBS and resuspended in 50 μl PBS. The particles in the resuspended pellet were analyzed by tunable resistive pulse sensing (TRPS, qNano, Izon Sciences) [27]. The protein content of the sEV-enriched preparations was determined by the Micro-BCA assay (Pierce). The IFITM3 content was determined by a commercial ELISA kit (MyBiosource).

### Flow cytometry analysis of EPCs and sEVs

BM cells were stained with PE-conjugated anti-CD34-PE and anti-VEGFR2-PerCP/Cy5.5 antibodies (Sony). Blood samples were collected into EDTA tubes, red blood cells were lysed, and anti-CD34-PE and CD31-FITC (Sony) as well as an isotype control (rat IgG2a, Sony) were used to detect EPCs. Labeled cells were detected with a flow cytometer (FACSCalibur, BD Bioscience), and analysis was performed using the CellQuest and FlowJo softwares.

For sEV analysis, EV-enriched pellet was incubated with 1 μg of 4 μm aldehyde/sulfate latex beads (Invitrogen) for 15 min in 50 μl of 0.9% NaCl-HEPES puffer followed by an overnight incubation at 4 °C with agitation. The reaction was stopped by incubation with 100 mM glycine for 30 min at room temperature. sEV- or BSA-coated beads were washed with 1% PBS-BSA, blocked with 0.2% Tropix I-Block (Thermo) and incubated with either anti-CD81-PE (BD Bioscience) or isotype control (Armenian hamster IgG2), anti-CD63-PE (BioLegend), anti-CD107a PerCP/Cy5.5 (LAMP2, Sony), anti-CD9-PE (Abcam), isotype control (rat IgG2a) or anti-IFITM3 (ProteinTech), and a goat anti-rabbit IgG-Cy2 (Abcam) in PBS–BSA 1% for 30 min at 4 °C. Next, the samples were washed and analyzed on a FACSCalibur flow cytometer (BD Bioscience).

### In vivo treatment of mice with GW4869

C57BL/6 mice (10 to 12 weeks of age) were randomly assigned to be injected with PBS, RD-LPS, or GW4869+RD-LPS (*n* = 5 per group). GW4869 (Sigma) was dissolved in DMSO (0.005%) and injected i.p. at a single dose of 25 μg/mouse. Mice in the GW4869+RD-LPS group were pre-injected i.p. with 20 μg RD-LPS 1 h prior to the i.p. injection of GW4869. Twenty-four hours later, the femurs were removed and BMCs were isolated.

### Transmission electron microscopy

The sEV pellets were fixed with 4% paraformaldehyde, washed with PBS, and post-fixed in 1% OsO_4_ for 30 min as previously published [28]. After rinsing with distilled water, pellets were dehydrated in graded ethanol, including block staining with 1% uranyl-acetate in 50% ethanol for 30 min, and were embedded in Taab 812. Overnight polymerization of samples at 60 °C was followed by sectioning and analysis using a Hitachi 7100 electron microscope (Hitachi Ltd).

### Protein identification using LC-MS (MS)

EV pellets were resuspended in 25 μL HPLC water and proteins were extracted as described previously [29]. The resulting peptides were desalted using Pierce™ C18 spin columns (Thermo). Peptides were analyzed using a Waters nanoACQUITY UPLC (Waters) coupled to a high-resolution maXis II QTOF mass spectrometer equipped with CaptiveSpray nanoBooster ionization source (Bruker). Peptides were separated using gradient elution on a 25-cm Waters Peptide BEH C18 nanoACQUITY 1.7 μm particle size UPLC column. Data were processed using proteinScape 3.0 software (Bruker). Proteins were identified using Mascot (version 2.5, Matrix Science) search engine against the Swissprot *Mus musculus* database (accessed on 09/2017). The following parameters were used: trypsin enzyme, 7 ppm peptide mass tolerance, 0.05 Da fragment mass tolerance, two missed cleavages. Carbamidomethylation was set as fixed modification, while deamidation (NQ) and oxidation (M) as variable modifications. Proteins with a minimum of two identified, unique peptides were accepted. Gene ontology enrichment was performed using g:Profiler [30]. Label-free quantification was performed using MaxQuant [31] software version 1.5.3.30. Each LC-MS/MS run was aligned using the “match between runs” feature (match time window 0.8 min, alignment time window 15 min).

### Silencing IFITM3 by lentiviral particle containing shRNA

The commercially available lentiviral particles (Santa-Cruz) were named as sh-IFITM3 or sh-control (scrambled). Recombinant lentiviral vectors expressing IFITM3 shRNA or control shRNA constructs at multiplicity of infection (MOI) = 50 were transduced in the presence of 8 μg/ml polybrene into freshly isolated BM cells. Knockdown of IFITM3 RNA was confirmed by qRT-PCR and on protein level with flow cytometry. Total RNA was extracted from BM cells 2 days after infection using the RNeasy Mini Kit (Qiagen). cDNA was synthesized using the SensiFAST cDNA Synthesis Kit (Bioline). Real-time quantitative PCR analysis was performed using the SYBR Green Master Mix Kit (Bioline) on a 7900HT real-time PCR platform (Thermo Fisher Scientific). For relative quantification, 2-^ΔΔCT^ was calculated. The primer sequences for PCR amplification of the IFITM3 gene were 5′-TGTCCAAACCTTCTTCTCTCC-3′ and 5′-CGTCGCCAACCATCTTCC-3′. GAPDH was applied as an internal control.

### Statistics

Survival rates were analyzed by the Kaplan-Meyer test. Data from > 3 groups were analyzed by non-repeated measures ANOVA with Dunnett’s test for comparison with the control. Unpaired two-tailed Student’s *t* test was used to analyze data between two groups. For all data analysis, we used GraphPad Prism 8.0.2.

## Results

### Radioprotective effect of RD-LPS

**Fig. 1:**
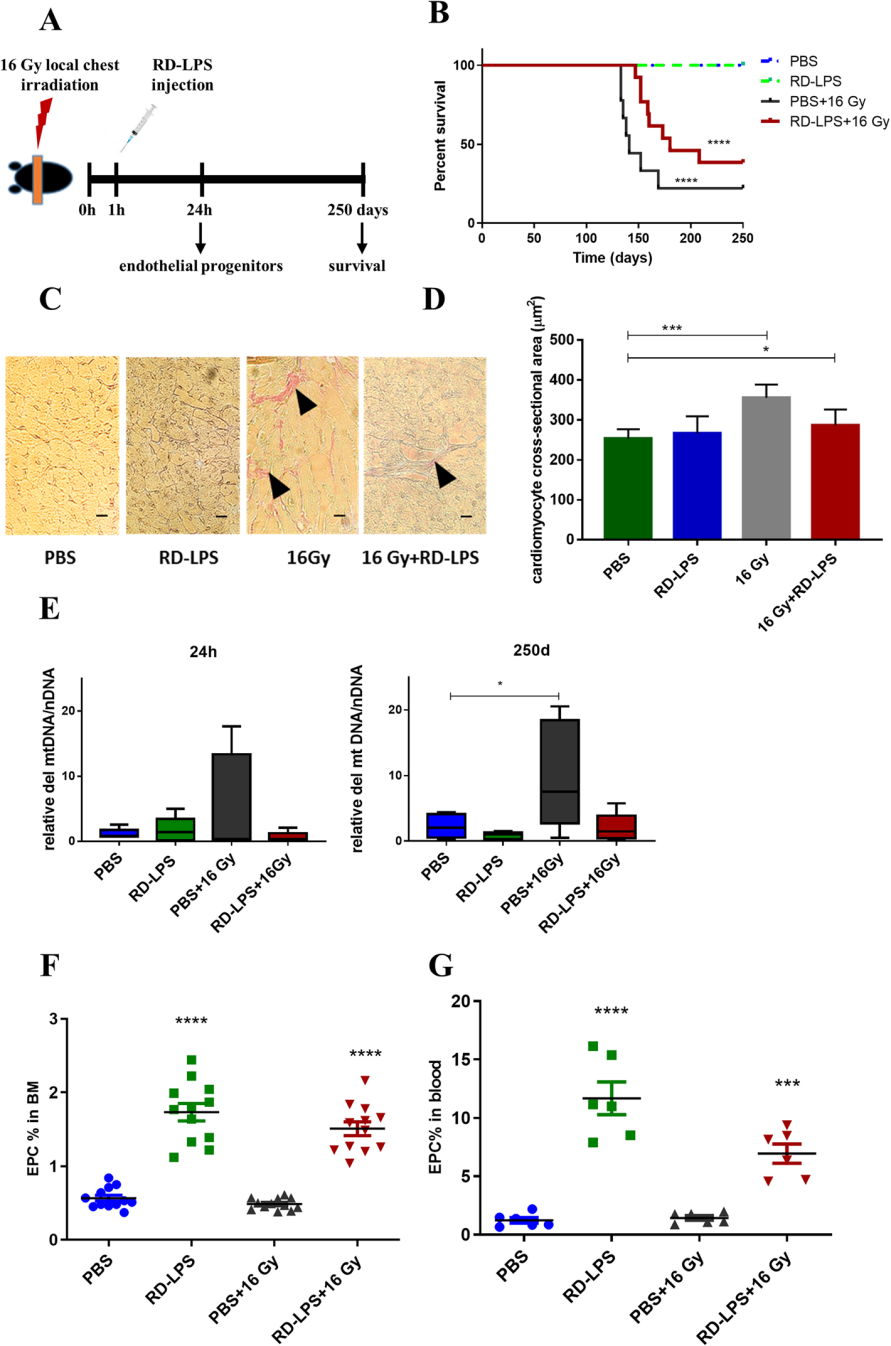
Effect of local chest irradiation and RD-LPS treatment. **a** A schematic illustration of the experimental setup. Hearts of C57BL/6 mice received a 16-Gy single dose of X-ray radiation. **b** Kaplan-Meyer diagram shows the changes of survival in animals treated with PBS, PBS+16 Gy of X-ray, RD-LPS (20 μg/mouse) or 16 Gy combined with RD-LPS (*n* = 9–10/group). *p* < 0.001, Log-rank Mantel-Cox test. **c** Representative images of histopathologic changes at 250 days after local heart irradiation. Interstitial fibrosis and degeneration of cardiomyocytes after 16 Gy are indicated (arrows). Van Gieson staining, × 20 objective magnification. Scale bar: 50 μm. **d** Morphometric analysis of cardiomyocyte cross-sectional area 250 days post-treatment. Original magnification × 20. Quantitative analysis of myocyte cross-sectional area is shown; *p < 0.05 and ***p < 0.005 vs. PBS-treated animals. **e** RD-LPS protects against the radiation-induced accumulation of deleted mitochondrial DNA. Copy number was evaluated with qPCR as detailed in “Materials and methods”. **f** Flow cytometry analysis of EPCs in the BMCs and **g** in the circulation. One-way ANOVA, **p < 0.01; ***p < 0.001, *n* = 6–12/group. Mean ± SD values are shown

In the present study, we addressed the question whether radio-detoxified LPS (RD-LPS) has a protective role if administered i.p. 1 h after local thorax irradiation (IR) of mice (Fig. 1a). As shown in Fig. 1b, most mice died between days 133–169 post-irradiation. The median survival was 141 days in the 16-Gy-irradiated group and 180 days in the irradiated and RD-LPS-treated mice (log-rank Mantel-Cox test, *p* < 0.001). In C57Bl/6 mice, the number of PBMCs decreased in the heart-irradiated groups 24 h, and 1 week post-irradiation, the number of BMCs decreased at 24 h in the RD-LPS+16 Gy groups and 1 week post-irradiation in the RD-LPS group (Additional file 1). The 16-Gy-treated animals developed bilateral ventricular hypertrophy as evidenced by gravimetry after 250 days, but RD-LPS protected against radiation-induced myocyte hypertrophy (Additional file 2). Hearts of irradiated and RD-LPS-treated mice were further examined for evidence of pathologic alterations [32]. Van Gieson staining showed slight interstitial fibrosis in the irradiated heart 250 days after 16 Gy and RD-LPS+16 Gy (Fig. 1c). Quantitative analysis of the images indicated a significant increase of myocyte size 250 days after 16 Gy (*p* < 0.001) and RD-LPS+16 Gy (*p* < 0.05) compared with control mice (Fig. 1d). Mitochondrial DNA deletion, as a molecular marker of cardiomyocyte damage was assessed [33]. The results showed that the formation of mtDNA deletion in early and late time points in the irradiated groups was higher than in the controls, but this difference was significant only at 250 days post-irradiation (*p* < 0.05). Preventive effect of RD-LPS was also detected (Fig. 1e).

### RD-LPS enhances the mobility and migration of EPCs

**Fig. 2:**
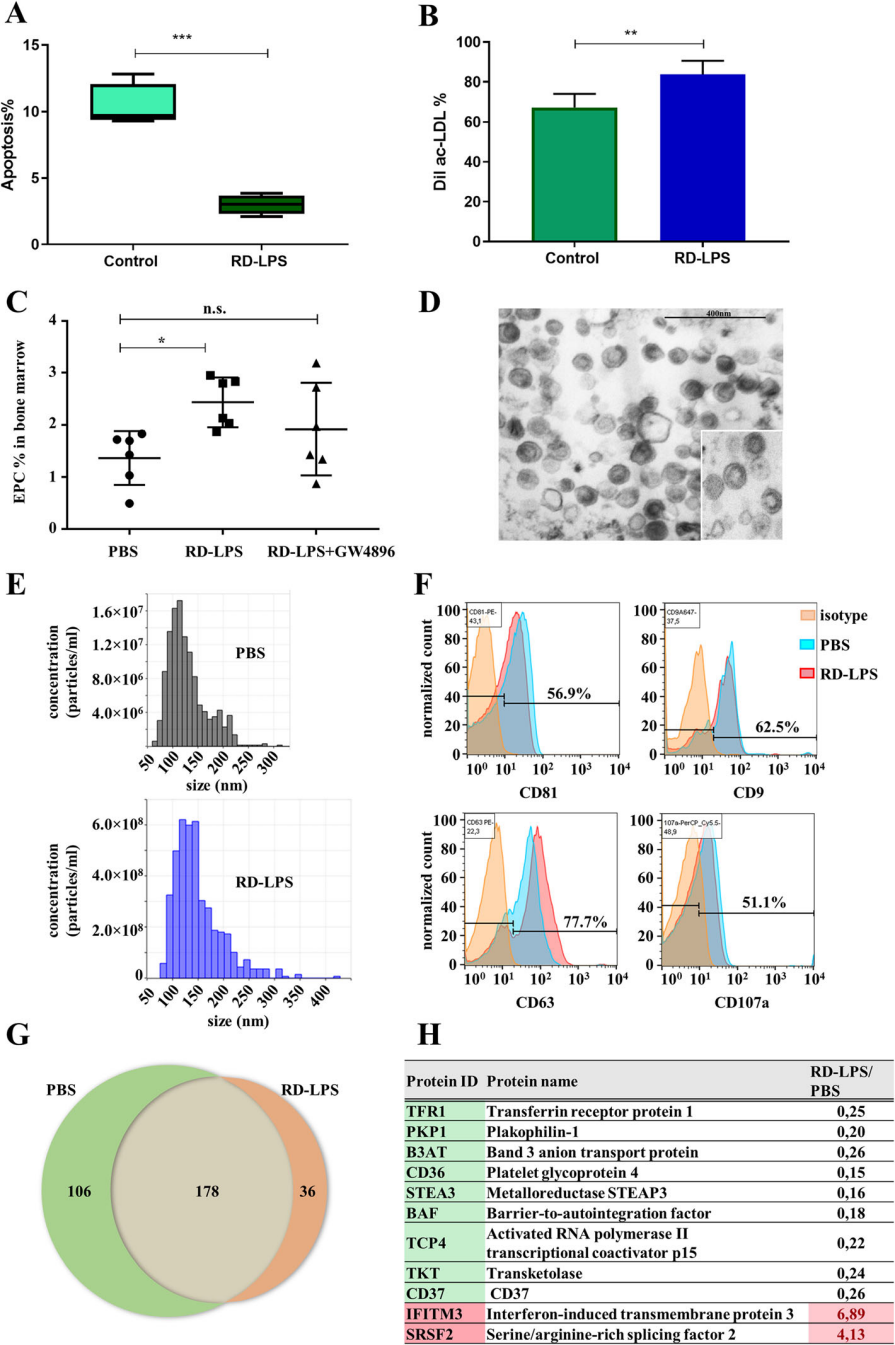
Effect of RD-LPS treatment viability and the function on EPCs. **a** RD-LPS protects against apoptosis of the lineage-negative stem cell population in the BM. BMCs were isolated, labeled with annexin V-FITC and To-Pro3, and measured by flow cytometry. **b** RD-LPS stimulates uptake of DiI-labeled ac-LDL by EPCs. Following 10 days of BMC culture in EBM-2 medium in the presence of vehicle PBS or 100 ng/ml RD-LPS, EPCs were incubated for 4 h with 1 μg protein/ml DiI-ac-LDL, as described in the “Materials and methods”. Cell-associated fluorescence was quantitated by flow cytometry. Each point represents the mean fluorescence of 10,000 cells. The experiment was repeated three times with similar results, *n* = 6, ***: *p* = 0.0002. *Investigation of the role of sEVs in* in vivo *RD-LPS response.* **c** RD-LPS-stimulated EPC response of the BM was abolished by in vivo GW4869 treatment (20 μg/mice), measured by flow cytometry (*n* = 5–6). One-way ANOVA, *p < 0.05 PBS vs. RD-LPS. Mean ± SD values are shown. **d**–**f** The characterization of sEVs isolated from the conditioned media of BMCs. **d** Representative transmission electron microphotographs (TEM) of sEVs isolated from the conditioned media of BMCs of PBS-injected (large image) and RD-LPS-injected mice (insert). **e** Particle size distribution and concentration in the sEV-enriched preparations isolated from the conditioned media of BMCs determined by tunable resistive pulse sensing (qNano). The upper and lower panels show representative profiles of sEVs in the PBS-injected and RD-LPS-injected groups, respectively. **f** Flow cytometry analysis of BMC-derived sEVs. Representative results of three independent experiments are shown. **g** Venn diagram of PBS control and RD-LPS-treated sEV proteome. The numbers of identified proteins are indicated. **h** Quantitative analysis of differentially expressed proteins in RD-LPS and PBS samples. Fold change threshold is > 4. Green- and pink-labeled proteins are down- and upregulated, respectively

Next, we set to examine the number of EPCs in the BM of experimental groups of mice. As shown in Fig. 1f, the number of EPCs was higher in the groups of mice injected with RD-LPS with or without irradiation as compared to the PBS-injected controls (*p* < 0.001 for both groups). We also analyzed EPCs in blood samples of mice and also found a significant difference between the above groups (*p* < 0.001 and *p* < 0.005, respectively, see Fig. 1g). Since RD-LPS stimulated the differentiation and also the release of EPCs into the circulation, we speculated that RD-LPS may promote the survival of BM stem cells and the uptake of ac-LDL by EPCs. To test this, isolated BMCs were treated with RD-LPS for 24 h and the apoptosis of lineage-negative stem cells were measured by flow cytometry. The proportion of early apoptotic cells were 10.39 ± 1.6% in the control group and 2.99 ± 0.7 in the RD-LPS group (*p* = 0.0002) as shown in Fig. 2a and Additional file 3, while late apoptosis (annexin V and TO-PRO positive cells) did not show significant differences (data not shown).

Next, BM cells were cultured in an endothelial induction medium (EGM2, Lonza) to obtain endothelial progenitor cells. To identify the resulting EPCs, we measured surface markers CD34 and VEGFR2 and the expression of endothelial specific mRNAs CD31 and VE-cadherin and also investigated the physiologic functions of these cells by monitoring DiI-labeled acetylated LDL (DiI-ac-LDL) endocytosis [34]. Following in vitro treatment with RD-LPS, EPCs were incubated for 4 h with DiI-ac-LDL and cell-associated fluorescence was analyzed by fluorescence microscopy (Zeiss Celldiscoverer, Additional file 4, panels A-C) and quantitated by flow cytometry. As shown in Fig. 2b, treatment with RD-LPS led to a significant increase in cell association (a combination of binding and uptake) of DiI-ac-LDL. To investigate the effects of RD-LPS on EPC, the biological factors associated with matured endothelial cells were measured by real-time RT-PCR. The expression of two markers (CD31 and VE-cadherin) were elevated in the RD-LPS-treated group as compared to the PBS group (Additional file 4, panels D-E). CD31 increased by 3.89 ± 0.5 fold, whereas VE-cadherin increased by 10.68 ± 3.8 fold.

### Effect of RD-LPS on BM-derived small extracellular vesicles

Subsequently, we examined whether blocking the generation of sEVs would modulate the RD-LPS-induced EPC response. To delineate the possible involvement of sEVs in the RD-LPS-induced changes in EPC numbers, we investigated the in vivo effects of GW4869. GW4869 is a neutral sphingomyelinase inhibitor reported to inhibit the ESCRT-independent sEV biogenesis [35]. Indeed, GW4869 attenuated the RD-LPS-induced increase in EPC number in BM (Fig. 2c).

To confirm that BM cells secrete RD-LPS-induced sEVs, we isolated sEVs from primary BMCs. Transmission electron microscopy (TEM) confirmed the presence and morphology of the sEVs (Fig. 2d). Next, we analyzed the particle size distribution and concentration of our sEV-enriched preparations by TRPS. Figure 2e shows representative size distribution of EVs from BMCs isolated from control (upper histogram) and RD-LPS-injected mice (lower histogram) which corresponded to the size range of sEVs and did not differ between the experimental groups. Of note, RD-LPS induced an order of magnitude higher number of EVs as compared to PBS controls (Fig. 2e). Using flow cytometry, we confirmed the presence of sEV markers (CD81, CD9, CD63, and CD107a) on our sEVs bound onto latex beads (Fig. 2f).

**Fig. 3:**
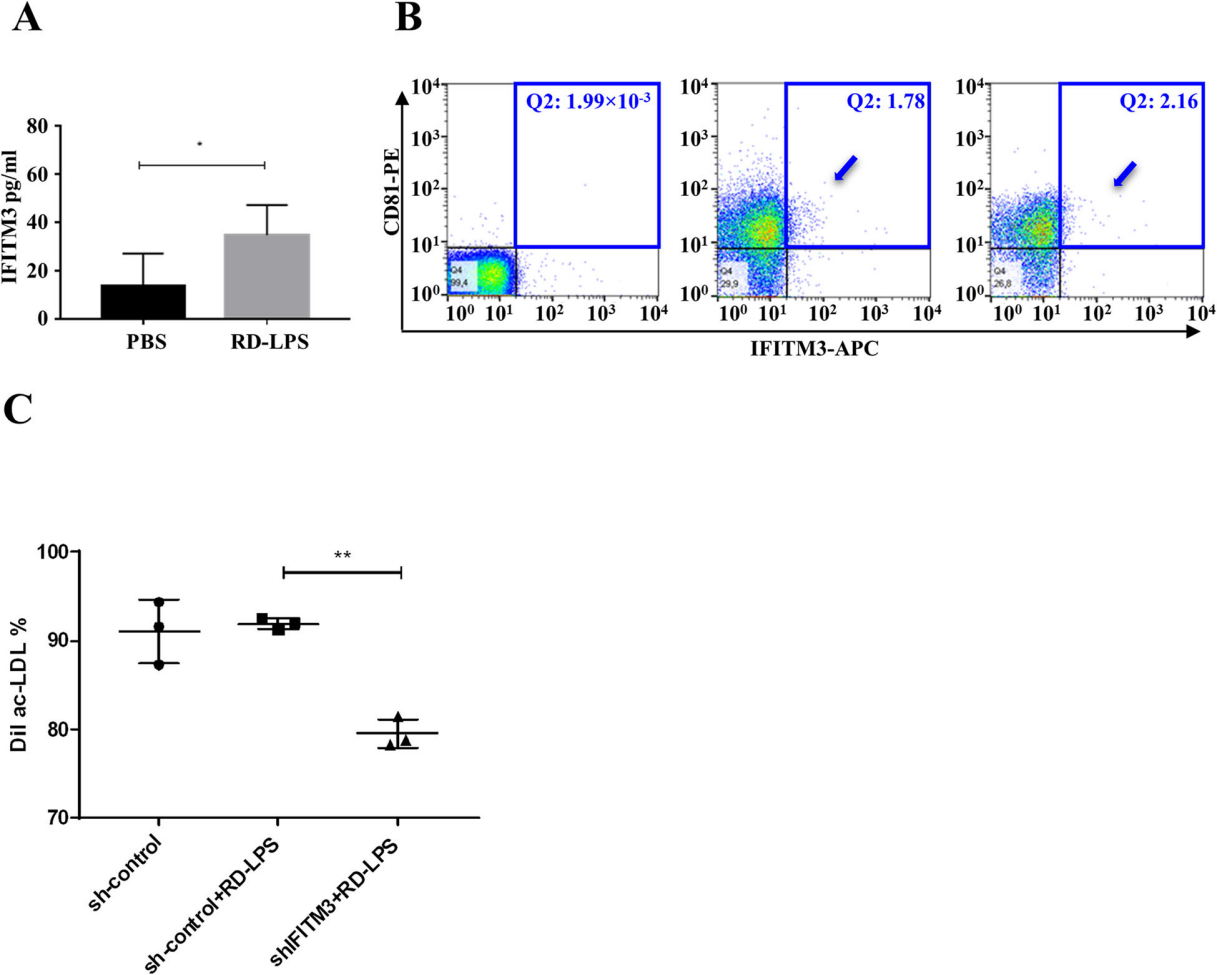
Validation of the expression and function of IFITM3 in BMC-derived sEVs. **a** Data (mean ± SD) measured using ELISA (*n* = 5). Unpaired Student’s *t* test, *p < 0.05. **b** BMC-derived sEVs from PBS- or RD-LPS-treated mice were stained with anti-CD81 and anti-IFITM3 and were analyzed by flow cytometry. Within Q2 quadrants, numbers indicate the relative percentages of positive cells. Double (CD81 and IFITM3) positive sEVs are shown by arrows. Left: isotype control, middle: PBS-injected group and right: RD-LPS group. **c** Loss of IFITM3 reduces BM-derived sEV-mediated modulation of function of EPCs. Differentiated EPCs were treated with the conditioned medium either of sh-control-transduced BMCs or sh-control-transduced and RD-LPS-treated BMCs or sh-IFITM3-transduced and RD-LPS-treated BMCs. DiI-ac-LDL uptake was measured by flow cytometry, the measurements were repeated three times, and the panel shows mean ± SD, **p < 0.01

Next, to address the question which proteins could play a role in the function of RD-LPS, we performed mass spectrometry analysis of the isolated sEVs. We identified 320 proteins in sEVs. Among them, 178 were shared by EVs isolated from the conditioned medium of BMCs from PBS-injected and RD-LPS-injected animals (Fig. 2g, Additional file 5). We classified the identified proteins using gene ontology (GO), based on biological process, molecular process, and cellular function. The sEV proteins were mainly involved in angiostatin binding (GO:0043532) and positive regulation of blood vessel endothelial cell migration (GO:0043536) (Additional file 6). Cellular components of the identified proteins revealed that most proteins were related to EVs (GO:1903561) (Additional file 6). We identified IFITM3 as a protein that showed the highest (6.89×) fold change (Fig. 2h). Using an ELISA system, we validated this MS finding (Fig. 3a). The expression of IFITM3 was low in BMC sEVs of the PBS-injected group, while it was enriched in sEVs in the RD-LPS-injected mice (*p* > 0.05). Similar results were measured from cells (data not shown). Figure 3b shows that flow cytometry confirmed double (IFITM3 and CD81) positivity of the sEV-enriched preparation.

### Effect of IFITM3 silencing on the activation of EPCs by RD-LPS

The role of vesicular IFITM3 on RD-LPS-induced EPCs was assessed by RNA silencing experiments. As shown in Additional file 7, high expression levels of IFITM3 were observed in RD-LPS-treated BMCs in vitro. To verify that the IFITM3 gene was silenced by sh-IFITM3, we determined the mRNA levels (Additional file 7A) in sh-control (scrambled control) and sh-IFITM3-infected cells by qRT-PCR. Cells infected with sh-IFITM3 exhibited significantly decreased IFITM3 mRNA levels, showing a 69.8 ± 7.5% reduction. To confirm the silencing of IFITM3, flow cytometry with IFITM3 antibodies was used. Compared with sh-control-infected cells, the IFITM3 protein level was significantly (89.2 ± 0.9%) decreased in BM cells infected with the sh-IFITM3 lentivirus (Additional file 7B). To determine whether IFITM3 has any impact on the RD-LPS’s action, EPC number was measured by flow cytometry and ac-LDL uptake was analyzed in differentiated EPCs by fluorescent microscopy and quantified by flow cytometry. Our data indicated that depletion of IFITM3 abolished the RD-LPS induction on EPCs as compared to RD-LPS-treated sh-control cells (2.77 ± 1.26% vs. 4.20 ± 0.79%, see Additional file 8), suggesting that the reduced expression of IFITM3 could significantly inhibit the elevation of EPCs in BM. Reduction of IFITM3 blocked the ac-LDL uptake in RD-LPS-treated cells compared to sh-control cells (75.65 ± 0.8% vs. 81.18 ± 1.6%, see Additional file 9). These results indicate that silencing of IFITM3 by RNAi could inhibit the effect of RD-LPS on induction of IFITM3 in BM cells.

Finally, to address the question if the IFITM3 cargo in sEVs could play a role in the effect of RD-LPS, we treated differentiated EPCs with the conditioned medium of sh-IFITM3-transduced BM cells. Acetylated LDL uptake was quantified in differentiated EPCs with flow cytometry (Fig. 3c). Adding conditioned medium of RD-LPS-treated BM cells to naive EPCs did not induce the further uptake of ac-LDL in sh-control+RD-LPS cells compared with sh-control cells (92.3 ± 0.4% vs. 91.2 ± 4.6%). However, downregulation of IFITM3 suppressed the activity of EPCs in sh-IFITM3+RD-LPS groups to 78.2 ± 0.8% (see Fig. 3c), indicating that IFITM3 modulates the function of differentiated EPCs.

**Fig. 4:**
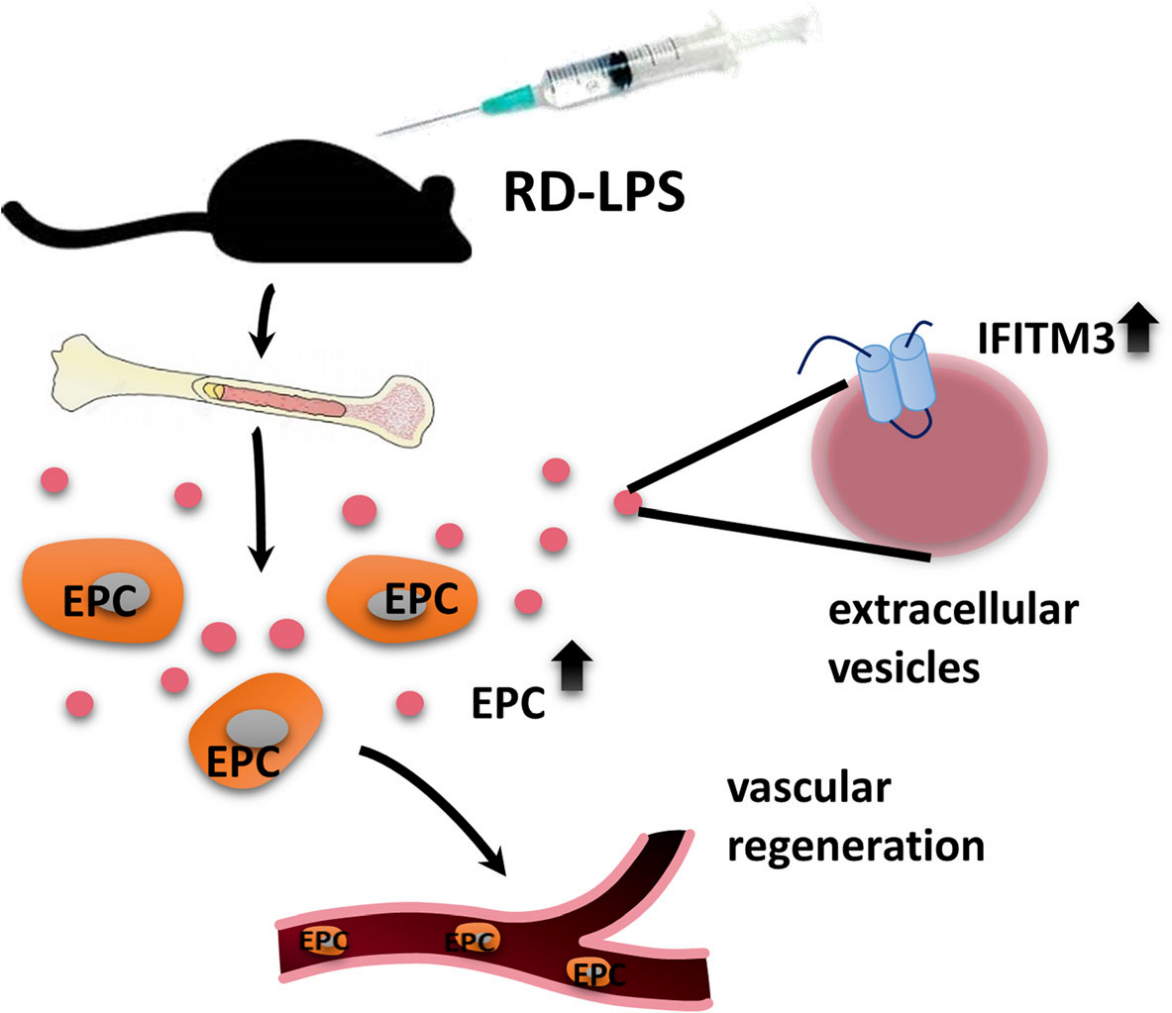
Overview of RD-LPS-induced release of bone marrow-derived small EVs with IFITM3 cargo. C57/Bl mice were injected i.p. with RD-LPS. After 24 h, the amount of EPCs (depicted as orange cells with gray nuclei) were elevated both in the bone marrow and peripheral blood, potentially playing a role in the vascular regeneration of distant damaged tissues. Small EVs (dark pink dots) isolated from bone marrow cells presented upregulated IFITM3 expression (light blue)

A graphical summary of the study is shown in Fig. 4.

## Discussion

Breast cancer radiotherapy causes myocardial damage and increases the risk of cardiovascular disease and atherosclerosis [36]. The pathogenesis of radiation-induced heart disease is not completely understood: since there is no specific treatment for these complications, a prevention strategy is required. Many ongoing pre-clinical studies aim to elucidate molecular and cellular mechanisms of cardiovascular damage, and their goal is to find potential targets for intervention of cardiotoxicity [37]. Our data are in line with a previous study [38] showing structural damage of the myocardium in C57BL/6 mice after local heart irradiation. Although radiation induced only modest changes in cardiomyocyte morphology and radiation-induced mitochondrial DNA deletion, these were mitigated by RD-LPS. However, a single local heart dose of 16 Gy resulted in reduced survival, and RD-LPS was protective against sudden death. Similar results were presented in dog models [39]. Since the endothelium of the vasculature is thought to be one target for injury induced by radiation, pharmaceutical approaches to prevent radiation-related cardiac injury present a potential method to mitigate and treat these types of damages. Not surprisingly, there has been intense interest in cardiac injury-mitigating therapeutic cell transplants; however, the major problems with this strategy have been the poor engraftment and retention of the administered cells [40]. Activating adult stem or progenitor cells may hold more promise as a therapeutic intervention strategy for vascular regeneration [41]. BM-derived EPCs have been implicated in the repair of irradiation-induced damage of capillaries via their mobilization into circulation [42]. In the present study, we aimed to investigate how RD-LPS impacts the release of EPCs from the BM into the circulation and if RD-LPS could play a protective role in irradiation-induced mortality of mice. We demonstrated association of high EPC count with reduced cardiac hypertrophy and mitochondrial DNA damage, which reinforces previous findings of their protective role in cardiovascular damage [43]. Our data suggest that in RD-LPS-treated mice EPCs proliferate and migrate, increasing their number in the circulation. Thus, RD-LPS treatment could have a future impact as an initiating factor triggering EPCs. In this model, we obtained the beneficial effect of RD-LPS on survival and the treatment-associated increase of the endogenous EPCs. Several pre-clinical models have demonstrated mobilization and migration of EPCs from the bone marrow niche followed by homing to the site of vascular damage, where they modulated vascular repair [44]. Endogenous factors used therapeutically, like granulocyte colony-stimulating factor (G-CSF) and GM-CSF, are also known to induce BM-EPC mobilization and migration but may present with further complications [45]. RD-LPS has no known adverse side effects and therefore may be a promising therapeutic agent for vascular regeneration.

In an earlier study, we have shown that a low-dose local head irradiation caused cerebrovascular damage and decreased the ability of endothelial repair by circulating progenitors [46]. In addition, in a mouse model of total body irradiation, administration of G-CSF improved the survival, presumably via promoting the self-renewal of hematopoietic stem cells [47]. Radioprotective action of RD-LPS has been confirmed in several in vitro and in vivo *models* [9, 48], but it has not been tested in cardiotoxicity mouse models yet. It is accepted that radiation damages the vascular endothelial cells [38] allowing the system-wide translocation of endotoxin from intestinal bacteria. The LPS then, among other adverse effects, induces inflammation associated with cardiotoxicity [49]. Here we found that radiation-detoxified LPS protects against radiation-induced lethality in 16-Gy-exposed mice and induced release of EPCs. Indeed RD-LPS reduced the apoptosis of the stem cells in the BM and induced the differentiation of the EPC culture in vitro. One of the possible mechanisms of the life-extending beneficial effects of RD-LPS could be that it antagonizes radiation-induced adverse effects of LPS by blocking its receptor.

The ability of isolated EPCs to uptake acetylated low-density lipoprotein (DiI-ac-LDL), the classical way to define endothelial cells, was performed as described earlier [50]. Two types of EPCs can be distinguished at different time points during in vitro differentiation: early and late EPCs [51]. EPCs can take up DiI-ac-LDL and express various combinations of surface markers of the endothelial cell lineage [52]. Our cells were late EPCs with the expressed matured endothelial-specific markers. EPCs at 10 days after plating also show the cobblestone morphology typical of endothelial cells (Additional file 4, panel C).

RD-LPS induced the number of sEVs, and we hypothesized that extracellular vesicles released from different cells are putatively involved in different aspects of the systemic response to RD-LPS, including paracrine and bystander effects. EVs may also act as stable reservoirs of biomarkers by containing specific cargo that reflect the effect of RD-LPS. We also investigated the effects of RD-LPS in vivo on the proteomic composition of BMC-derived sEVs [53] and observed pronounced differences in the protein profiles of BMC- and RD-LPS-injected BMC-derived sEVs. RD-LPS-induced sEVs carried a distinct set of proteins. Among them, IFITM3 showed the highest fold change as compared to the controls, validated also by an IFITIM3 ELISA and flow cytometry analysis of sEVs. This is in line with recent findings reporting IFITM3 in sEVs mediating paracrine senescence [54]. IFITM3 is best known to play a role in the defense against virus invasion and potentially inhibits EV fusion with cells [55, 56]. Small extracellular vesicle-associated IFITM3 has been also shown earlier to transfer an anti-viral effect to recipient cells [57]. Interestingly, IFITIM3 is present in migratory primordial germ cells and serves as a homing signal enabling cells to respond to environmental signals guiding their migration [58]. Recently, IFITIM3 has also been reported as a biomarker of several tumors [59, 60]. Moreover, IFITM3 was detected in endothelial cells and several cancer cell-derived sEVs by MS [61, 62]. We speculated that IFITM3 was necessary for the activation of differentiated EPCs and RD-LPS-induced EPC activation based on an early stem cell induction phase. Surprisingly, in our in vitro experiments, the RD-LPS effect was not transmitted through EVs. However, our data suggest that the presence of IFITM3 in the cells is necessary for RD-LPS-induced EPC activation, in agreement with a recent study pinpointing the possible role of IFITM3 in glioma cell growth and migration [63].

Our results suggest that the expression of the transmembrane protein IFITM3 is strongly upregulated in RD-LPS-treated mice. In addition, IFITIM3 is associated with BMC-derived sEVs and may provide a promising candidate biomarker of the RD-LPS effect. Thus, assessment of cellular and EV-associated IFITIM3 may possibly find a place among strategies for evaluating the regeneration potential of BM.

## Supplementary information

**Additional file 1.** The effect of RD-LPS injection on the count of peripheral blood mononuclear cells (PBMCs) and bone marrow cells (BMCs) of 16 Gy local heart-irradiated mice. Results of time dependent alterations of number of PBMCs and BMCs following irradiation and RD-LPS treatment.

**Additional file 2.** RD-LPS mitigates irradiation-induced cardiac hypertrophy. Irradiation-stimulated increase in heart weight at 250 days is abrogated by the addition of RD-LPS.

**Additional file 3.** RD-LPS reduces apoptosis of Lin- stem cells. The representative figures show the gating strategies.

**Additional file 4.** RD-LPS stimulates uptake of Dil-ac-LDL by EPCs. The representative images show the DiI-ac-LDL uptake.

**Additional file 5 **List of small extracellular vesicle proteins identified by LC-MS/MS in BM-derived cells. List of proteins identified by LC-MS/MS are given in separate sheets. Sheet ‘PBS’ shows the result of measurement on controls (*n*=7); the results of the treated group (*n*=9) are listed on sheet ‘RD-LPS’.

**Additional file 6.** Detailed results of the enrichment analysis. Output from the g:Profiler enrichment analysis for the three main Gene Ontology categories (Biological Process, Cellular Component, Molecular function).

**Additional file 7.** Lentivirus-mediated gene silencing of IFITM3 in BM cells. Results of qRT-PCR (A) and flow cytometric evaluation (B).

**Additional file 8.** Effect of lentivirus-mediated gene silencing of IFITM3 on EPC activation in BMCs. Flow-cytometry based measurement of the number of EPCs.

**Additional file 9.** Effect of lentivirus-mediated gene silencing of IFITM3 on the function of differentiated EPCs. Results of DiI-ac-LDL analyzed by flow-cytometry (A) and representative images (B).

## Data Availability

All data generated or analyzed during this study are included in this published article and its supplementary information files.

## Abbreviations

RD-LPS: Radio-detoxified lipopolysaccharide
sEV: Small extracellular vesicles
IFITM3: Interferon-induced transmembrane protein-3
BM: Bone marrow
BMCs: Bone marrow cells
EPCs: Endothelial progenitor cells
VEGFR-2: Vascular endothelial growth factor receptor 2
GW4869: 2-Propenamide, 3,3′-(1,4-phenylene)bis[N-[4-(4,5-dihydro-1H-imidazol-2-yl)phenyl]-, hydrochloride
LC-MS: Liquid chromatography/mass spectrometry
TEM: Transmission electron microscopy
G-CSF: Granulocyte colony-stimulating factor
