# Supplementary material for: Radio-detoxified LPS alters bone marrow-derived extracellular vesicles and endothelial progenitor cells
Source: Stem Cell Res Ther. 2019 Oct 29;10:313. doi: 10.1186/s13287-019-1417-4 (PMC6819448; doi:10.1186/s13287-019-1417-4)
Supplement: Supplementary file 1 — Additional file 1. The effect of RD-LPS injection on the count of peripheral blood mononuclear cells (PBMCs) and bone marrow cells (BMCs) of 16 Gy local heart-irradiated mice. Results of time dependent alterations of number of PBMCs and BMCs following irradiation and RD-LPS treatment. [file 13287_2019_1417_MOESM1_ESM.docx]

**The effect of RD-LPS injection on the count of peripheral blood mononuclear cells (PBMC/ and bone marrow (BM/ cells of 16 Gy local heart irradiated mice.**

|  | **24h** | | **7d** | | **250d** | |
| --- | --- | --- | --- | --- | --- | --- |
| **PBMC** | Mean ± SD (count/µl) | p* | Mean ± SD (count/µl) | p* | Mean ± SD (count/µl) | p* |
| PBS | 4.856 ± 1.612 × 10^3^ | -- | 4.650 ± 1.213 × 10^3^ | -- | 3.651 ± 0.930 × 10^3^ | -- |
| RD-LPS | 3.821 ± 1.455 × 10^3^ | ns. | 5.200 ± 1.129 × 10^3^ | ns. | 3.743 ± 1.141 × 10^3^ | ns. |
| PBS+16Gy | 1.807 ± 0.262 × 10^3^ | **<0.0001** | 3.179 ± 0.920 × 10^3^ | **<0.01** | 3.674 ± 1.612 × 10^3^ | ns. |
| RD-LPS+16Gy | 2.006 ± 0.682 × 10^3^ | **<0.0001** | 3.321 ± 0.608 × 10^3^ | **<0.05** | 4.061 ± 1.238 × 10^3^ | ns. |
|  |  |  |  |  |  |  |
| **BMC** | Mean ± SD (count/µl) | p | Mean ± SD (count/µl) | p | Mean ± SD (count/µl) | p |
| PBS | 3.248 ± 0.811 × 10^3^ | -- | 3.758 ± 0.920 × 10^3^ | -- | 4.103 ± 0.820 × 10^3^ | -- |
| RD-LPS | 2.687 ± 0.841 × 10^3^ | ns. | 2.473 ± 0.830 × 10^3^ | **<0.001** | 3.860 ± 1.207 × 10^3^ | ns. |
| PBS+16Gy | 3.030 ± 0.512 × 10^3^ | ns. | 2.990 ± 0.420 × 10^3^ | ns. | 4.520 ± 0.990 × 10^3^ | ns. |
| RD-LPS+16Gy | 2.336 ± 0.320 × 10^3^ | **<0.005** | 4.313 ± 1.541 × 10^3^ | ns. | 3.847 ± 1.085 × 10^3^ | ns. |
| *: ns. = not significant | |  |  |  |  |  |
|  | |  |  |  |  |  |

The PBMC and BMC profile of the mice on day 1, 7 and 250 after the indicated treatment are shown.

n= 6-12/group. Mean ± SD values are shown.
