# Supplementary material for: Radio-detoxified LPS alters bone marrow-derived extracellular vesicles and endothelial progenitor cells
Source: Stem Cell Res Ther. 2019 Oct 29;10:313. doi: 10.1186/s13287-019-1417-4 (PMC6819448; doi:10.1186/s13287-019-1417-4)
Supplement: Supplementary file 2 — Additional file 2. RD-LPS mitigates irradiation-induced cardiac hypertrophy. Irradiation-stimulated increase in heart weight at 250 days is abrogated by the addition of RD-LPS. [file 13287_2019_1417_MOESM2_ESM.docx]

**RD-LPS mitigates the irradiation-induced cardiac hypertrophy**

Total body weight and heart weight were measured by gravimetry, at 250 days after irradiation. Significance levels: * = p<0.05, *** = p<0.005, n= 9/group. Mean ± SD values are shown.
