# Supplementary material for: Radio-detoxified LPS alters bone marrow-derived extracellular vesicles and endothelial progenitor cells
Source: Stem Cell Res Ther. 2019 Oct 29;10:313. doi: 10.1186/s13287-019-1417-4 (PMC6819448; doi:10.1186/s13287-019-1417-4)
Supplement: Supplementary file 3 — Additional file 3. RD-LPS reduces apoptosis of Lin- stem cells. The representative figures show the gating strategies. [file 13287_2019_1417_MOESM3_ESM.docx]

**RD-LPS reduces apoptosis of Lin- stem cells**


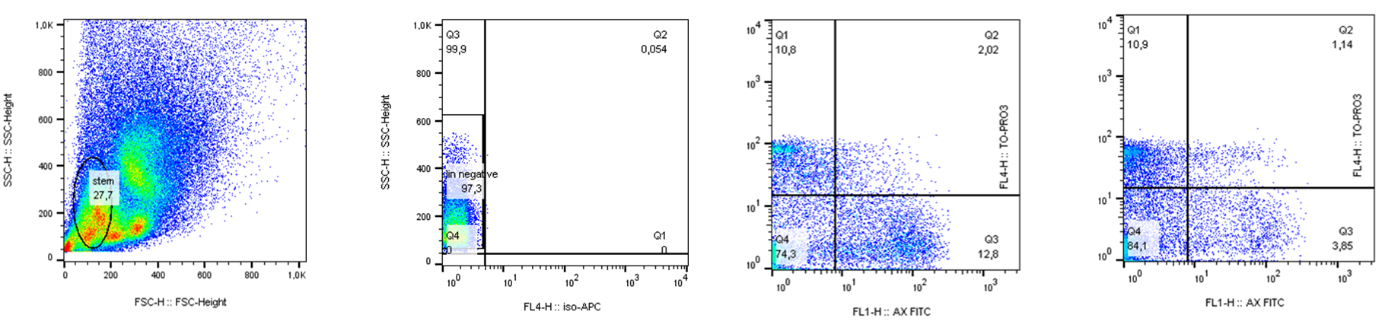


Analysis of apoptosis prevented by RD-LPS. Isolated BM cells were labeled with Annexin V –FITC (Sony) and 1 mg/μl To-Pro3 (Life Technologies) and measured by FACSCalibur. The representative figures show the gating strategies.
