# Supplementary material for: Radio-detoxified LPS alters bone marrow-derived extracellular vesicles and endothelial progenitor cells
Source: Stem Cell Res Ther. 2019 Oct 29;10:313. doi: 10.1186/s13287-019-1417-4 (PMC6819448; doi:10.1186/s13287-019-1417-4)
Supplement: Supplementary file 4 — Additional file 4. RD-LPS stimulates uptake of Dil-ac-LDL by EPCs. The representative images show the DiI-ac-LDL uptake. [file 13287_2019_1417_MOESM4_ESM.docx]

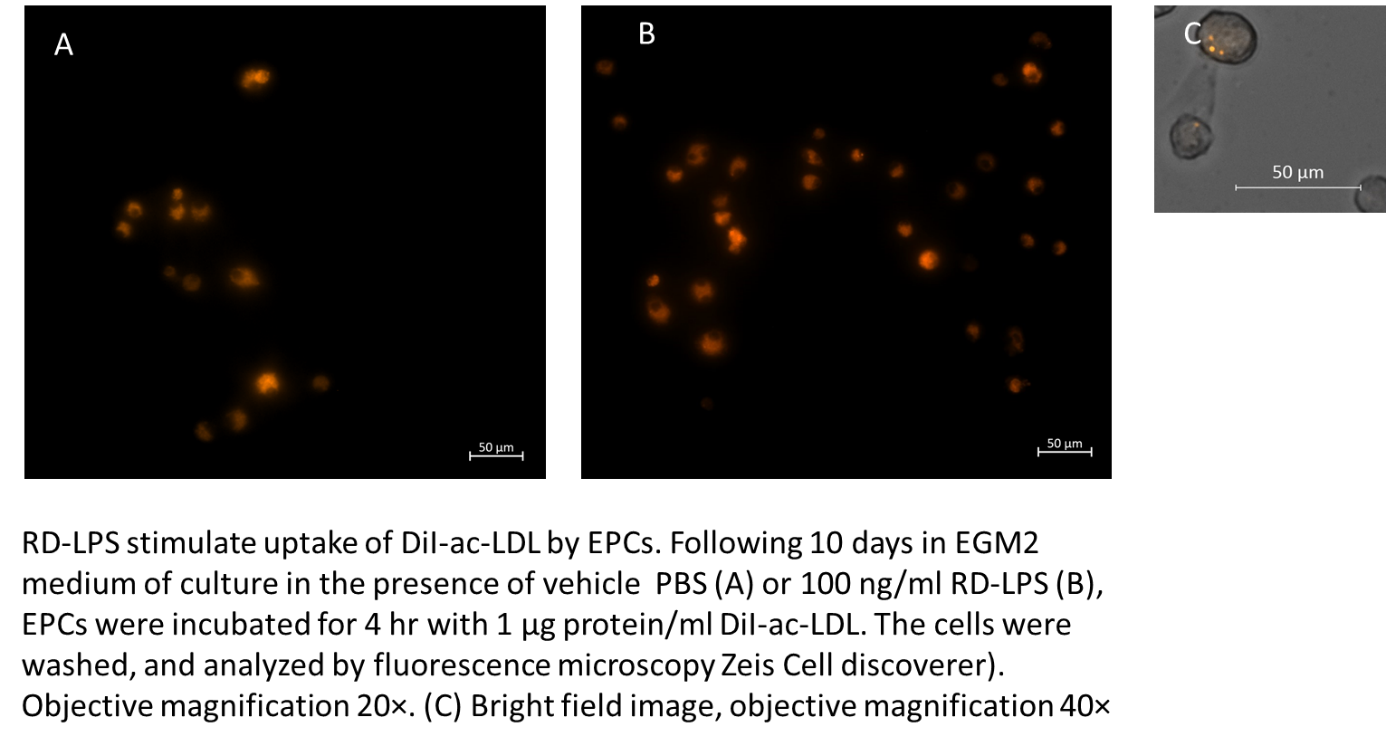
**RD-LPS stimulates uptake of Dil-ac-LDL by EPCs.**

**C PBS RD-LPS**

**
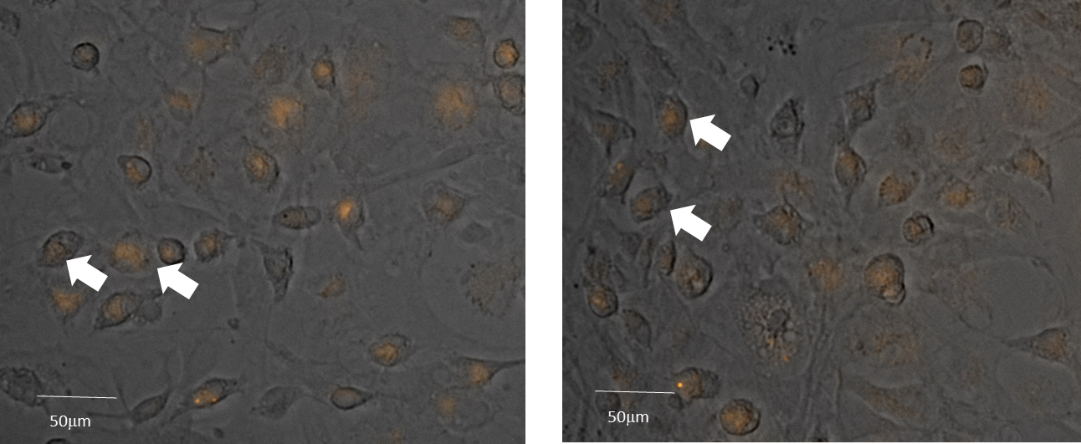
**

**RD-LPS increases expression** of **CD31 and VEGFR2 mRNA in differentiated EPCs**

Following 10 days in EGM-2 medium supplemented with EGM -2 SingleQuot (Lonza) in the presence of vehicle PBS (A,) or 100 ng/ml RD-LPS (B), , EPCs were incubated for 4 hours with 1 µg protein/ml DiI-ac-LDL. The cells were washed and analysed by fluorescence microscopy (Zeis Celldiscoverer). Objective magnification was 20x. (C) Bright field image, objective magnification 40x.

(D, E) Quantitative RT-PCR analysis of differentiated EPCs, corrected for expression of the control gene GAPDH. Of the endothelial genes, CD31 showed a significantly higher expression in RD-LPS treated cells (D). Expression levels of the VE-cadherin was also elevated in EPCs (E).

n = 4. *P < 0.05 and ***P < 0.001.

Data are shown as mean ± SD. Analysis was performed using two-tailed Student’s t-test.

Total RNA of differentiated-EPCs was prepared with the RNeasy Mini kit (Qiagen). Reverse transcription-polymerase chain reaction (RT-PCR) was performed by using the SensiFAST cDNA Synthesis Kit (Bioline) using 1 μg of total RNA. PCR amplification was performed with synthetic gene-specific primers for VE-cadherin (forward primer, 5'-AGATTCACGAGCAGTTGGTCA-3'; reverse primer, 5'-GATGTCAGAGTCGGAGGAATT-3'), for CD31 (forward primer, 5'-AGGCTTGCATAGAGCTCCAG-3’; reverse primer, 5'-TTCTTGGTTTCCAGCTATGG-3′) by using HT7900 cycler (Applied Biosystem). To quantify transcripts, quantitative RT-PCRs were performed and normalized to GAPDH expression. QPCR reactions are carried out using the SYBR Green PCR kit (Bioline). The fold change of each target gene’s mRNA was calculated relative to the control cells using the 2^-ΔΔCt^ formula.
