# Supplementary material for: Radio-detoxified LPS alters bone marrow-derived extracellular vesicles and endothelial progenitor cells
Source: Stem Cell Res Ther. 2019 Oct 29;10:313. doi: 10.1186/s13287-019-1417-4 (PMC6819448; doi:10.1186/s13287-019-1417-4)
Supplement: Supplementary file 7 — Additional file 7. Lentivirus-mediated gene silencing of IFITM3 in BM cells. Results of qRT-PCR (A) and flow cytometric evaluation (B). [file 13287_2019_1417_MOESM7_ESM.docx]

**Lentivirus-mediated gene silencing of IFITM3 in BM cells.**

**A B**

(A) Expression analysis of IFITM3 mRNA by qRT-PCR in cells infected with sh-control, and cells infected with sh-IFITM3 treated with or without RD-LPS. The GAPDH gene is the internal controls for qRT-PCR. Significant difference was found from sh-control compared with sh-control+RD-LPS and sh-IFITM3 (***=p<0.001, *=p<0.05). (B) Expression analysis of IFITM3 protein in cells infected with sh-control, and cells infected with sh-IFITM3 treated with or without RD-LPS group by flow cytometry. Data are expressed as Mean±SD n=3.
