# Supplementary material for: Radio-detoxified LPS alters bone marrow-derived extracellular vesicles and endothelial progenitor cells
Source: Stem Cell Res Ther. 2019 Oct 29;10:313. doi: 10.1186/s13287-019-1417-4 (PMC6819448; doi:10.1186/s13287-019-1417-4)
Supplement: Supplementary file 8 — Additional file 8. Effect of lentivirus-mediated gene silencing of IFITM3 on EPC activation in BMCs. Flow-cytometry based measurement of the number of EPCs. [file 13287_2019_1417_MOESM8_ESM.docx]

**Effect of lentivirus-mediated gene silencing of IFITM3 on EPC activation in BMCs.**

Presence of EPCs in BM cells infected with sh-control, and in cells infected with sh-IFITM3 treated with or without RD-LPS as analysed by flow cytometry. Data are expressed as Mean±SD n=3. *=p<0.05
