# Supplementary material for: Radio-detoxified LPS alters bone marrow-derived extracellular vesicles and endothelial progenitor cells
Source: Stem Cell Res Ther. 2019 Oct 29;10:313. doi: 10.1186/s13287-019-1417-4 (PMC6819448; doi:10.1186/s13287-019-1417-4)
Supplement: Supplementary file 9 — Additional file 9. Effect of lentivirus-mediated gene silencing of IFITM3 on the function of differentiated EPCs. Results of DiI-ac-LDL analyzed by flow-cytometry (A) and representative images (B). [file 13287_2019_1417_MOESM9_ESM.docx]

**Effect of lentivirus-mediated gene silencing of IFITM3 on the function of differentiated EPC**

**A**

**B**

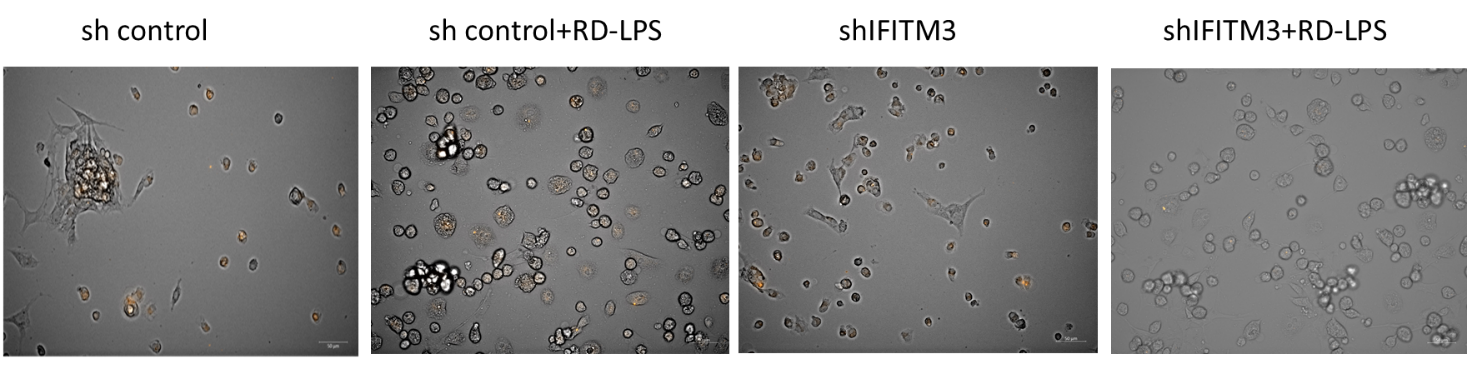

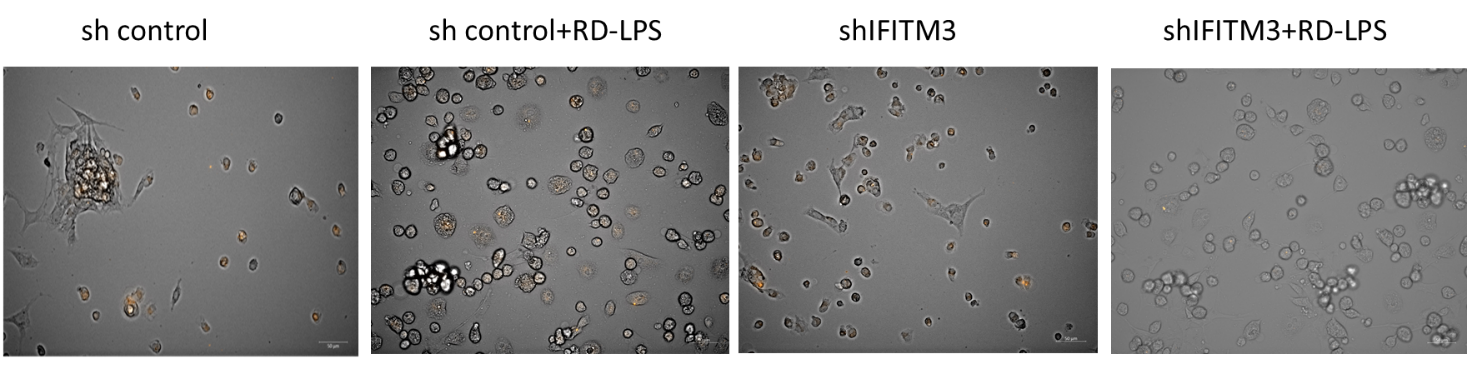


DiI-ac-LDL uptake was measured following 10 days in EGM2 culturing medium in the presence of vehicle PBS or 100 ng/ml RD-LPS and transduced with lentivirus sh-control or lentivirus sh-IFITM3. EPCs were incubated for 4 hours with 1 µg protein/ml Dil-ac-LDL. The cells were washed and analysed by flow cytometry. ***= p<0.001, **=p<0.005 (A). Panel (B) shows representative images taken by fluorescence microscopy (Zeis Celldiscoverer). Objective magnification was 20x.
